# Co-expression of Arabidopsis *NHX1* and *bar* Improves the Tolerance to Salinity, Oxidative Stress, and Herbicide in Transgenic Mungbean

**DOI:** 10.3389/fpls.2017.01896

**Published:** 2017-11-02

**Authors:** Sanjeev Kumar, Angkana Kalita, Richa Srivastava, Lingaraj Sahoo

**Affiliations:** Department of Bioscience and Bioengineering, Indian Institute of Technology Guwahati, Guwahati, India

**Keywords:** salt tolerance, herbicide resistance, oxidative stress tolerance, vacuolar Na^+^/H^+^ antiporter, *AtNHX1*, Na^+^ compartmentalization, mungbean

## Abstract

Mungbean is an important pulse crop extensively cultivated in Southeast Asia for supply of easily digestible protein. Salinity severely limits the growth and productivity of mungbean, and weeding poses nutritional and disease constraints to mungbean cultivation. To pyramid both salt tolerance and protection against herbicide in mungbean, the *AtNHX1* encoding tonoplast Na^+^/H^+^ antiporter from Arabidopsis, and *bar* gene associated with herbicide resistance were co-expressed through *Agrobacterium-mediated* transformation. Stress inducible expression of *AtNHX1* significantly improved tolerance under salt stress to ionic, osmotic, and oxidative stresses in transgenic mungbean plants compared to the wild type (WT) plants, whereas constitutive expression of *bar* provided resistance to herbicide. Compared to WT, transgenic mungbean plants grew better with higher plant height, foliage, dry mass and seed yield under high salt stress (200 mM NaCl) in the greenhouse. The improved performance of transgenic plants under salt stress was associated with enhanced sequestration of Na^+^ in roots by vacuolar Na^+^/H^+^ antiporter and limited transport of toxic Na^+^ to shoots, possibly by restricting Na^+^ influx into shoots. Transgenic plants showed better intracellular ion homeostasis, osmoregulation, reduced cell membrane damage, improved photosynthetic capacity, and transpiration rate as compared to WT when subjected to salt stress. Reduction in hydrogen peroxide and oxygen radical production indicated enhanced protection of transgenic plants to both salt- and methyl vialogen (MV)-induced oxidative stress. This study laid a firm foundation for improving mungbean yield in saline lands in Southeast Asia.

## Introduction

Mungbean (*Vigna radiata* L. Wilczek) is an important short duration grain legume, which provides an invaluable source of easily digestible protein diet lacking flatulence factors. It is well adapted to a large number of cropping systems worldwide and thus cultivated throughout Asia, Australia, South and North America, tropical and subtropical Africa (Ebert, 2014). India is the world's largest mungbean producer accounting for about 65% of world's acreage and 54% of global production (Singh and Singh, 2011). In recent years, the demand for mungbean production has increased significantly due to the domestic consumption and export potential, creating opportunities to enhance the revenue and livelihood of small land holding mungbean farmers. However, most of the cultivated mungbean genotypes are sensitive to salinity, which severely limits their yield potential under saline soil. Moreover, use of poor quality water for irrigation, poor drainage, or agricultural practices, and increased salinity of agricultural land further complicate mungbean production (Ghosh et al., 2015).

Growth and yield of mungbean in last decades is largely affected due to salinity stresses worldwide (Sehrawat et al., 2013). Incidence of more than 60% yield losses at 50 mM NaCl (Abd-Alla et al., 1998) has been reported accompanied with reduction in seed germination, fresh, and dry biomass, shoot and root lengths, photosynthesis, and yield attributes (Ghosh et al., 2015). In mungbean, the adverse effect of salt stress on grain yield (Minhas et al., 1990; Ayers and Westcot, 1994) is more at the reproductive stage than that at other stages. Salt stress induces ionic and osmotic imbalance and secondary oxidative stress that detrimentally affect the growth and metabolism of mungbean by disturbing the integrity of plant membrane, pigment content, osmotic adjustments, water retention capacity, and photosynthetic activity (Saha et al., 2010). Salt stress appears with physiological drought and nutritional deficiencies imposing attenuated toxicity on crop growth and metabolism, an issue that deserves major attention. It is estimated that increased salinity will cause an irresistible global effects, resulting ~50% loss of arable land by mid of the twenty-first century (Hasanuzzaman et al., 2012). Salinity stress adverse effects on plant growth, symbiotic association of nodules, and soil rhizobacteria for the nitrogen fixation. Ensuring food security for the increasing world population is nearly impossible without considerably increasing the crop production in such marginal areas (Nirmala et al., 2016). In this scenario, development of mungbean cultivars with improved salt tolerance and growth performance could increase the yield of this important grain legume and restore its productivity on salinity-affected agricultural lands.

Salt tolerance is a polygenic, highly intricate, and complex trait which firmly depends on genotype and plant developmental stages, thus developing salt stress tolerant varieties through traditional breeding approaches remain a difficult task. Conventional breeding using one or two traits has not yielded satisfactory result due to lack of key genes underlying the QTLs, impeding the success in genetic improvement of crops for salt tolerance. To overcome the limitations of classical breeding, transgenic approach is adopted for targeted improvement for salinity tolerance by introducing candidate gene(s) controlling salt-tolerant traits. Compartmentalization of Na^+^ into vacuoles by vacuolar Na^+^/H^+^ antiporter (*NHX*) in exchange of H^+^ (Apse et al., 1999), has long been proposed to play an important role in salt tolerance, by lowering deleterious effects of excess Na^+^ in the cytosol and maintaining osmotic balance in vacuoles using Na^+^ as an ionic osmolyte (Blumwald, 2000; Hasegawa et al., 2000; Adabnejad et al., 2015). In addition to salt stress response, pH homeostasis, these secondary antiporters are also involved in other biological processes including K^+^ homeostasis, cell expansion, and cellular vesicle trafficking (Tian et al., 2017). Reduction in plant growth and cell expansion across all tissues, and increased sensitivity to K^+^, in *Arabidopsis nhx1 nhx2* double mutant seedlings compared to either single mutant or wild-type plants clearly suggested the multiple physiological roles of *NHX* (Tian et al., 2017). First report on overexpression of vacuolar *NHX* of model plant Arabidopsis conferring salt tolerance was reported in transgenic tomato (Zhang and Blumwald, 2001). Since then, overexpression of vacuolar *NHX* conferring salt tolerance have been reported in a number of crop species (Rodríguez-Rosales et al., 2009), including *AtNHX1* in *Brassica napus* (Zhang et al., 2001), cotton (He et al., 2005), and buckwheat (Chen et al., 2008), *NHX1* homologs in Arabidopsis (Brini et al., 2007), rice (Fukuda et al., 2004; Zhao et al., 2006; Chen et al., 2007; Verma et al., 2007), wheat (Xue et al., 2004), barley (Fukuda et al., 2004), maize (Yin et al., 2004; Zörb et al., 2004; Chen et al., 2007), Indian mustard (Rajagopal et al., 2007), alfalfa (Li et al., 2011), cumin (Pandey et al., 2016), cowpea (Mishra et al., 2014, 2015), and industrial crops such as, tall fescue (Zhao et al., 2007), tobacco (Wu et al., 2004; Lu et al., 2005; Hossain et al., 2006; Zhang et al., 2008), Jatropha (Jha et al., 2013), castor (Patel et al., 2015), and switchgrass (Huang et al., 2017).

We recently reported enhanced salt tolerance in cowpea (Mishra et al., 2014) and mungbean (Sahoo et al., 2016) by constitutive expression of *VrNHX1* and *AtNHX1*, respectively. However, we observed while advancing the mungbean transgenic lines to subsequent generations, plant dwarfism and low yield possibly due to metabolic pay-offs and undesirable pleiotropic effects resulting from the constitutive expression of the transgene. Although expression of the heterologous genes through constitutive promoters has conferred abiotic stress tolerance in transgenic plants, it risks growth and yield even under low stress condition. Moreover, constitutive expression of stress tolerant genes is not desirable when plants do not encounter stress. On contrary, use of stress inducible promoters to drive the expression of these genes is potentially useful to get an optimal level of expression at the desired time and to avoid the unintended effects associated with constitutive expression on growth and yield under non-stressed conditions.

In addition to the salinity stress, weed competitions are one of the major constraints for mungbean productivity, with yield loss amounting to 65.4–79.0% (Shuaib, 2001; Dungarwal et al., 2003). Mungbean is sown as broadcast and therefore farmers do not practice weeding in mungbean field. The bar gene has been successfully used to develop transgenic plants with herbicide resistance (Fang et al., 2004; Manickavasagam et al., 2004; Sonia et al., 2007).

Therefore, in the present study, we decided to pyramid both salinity and herbicide tolerance, through stress inducible expression of *AtNHX1* and constitutive expression of *bar*. We report for the first time in mungbean, stacking of two desirable traits, tolerance to salinity and herbicide through co-expression of *AtNHX1* and *bar*, respectively.

## Materials and methods

### Vector construction and mungbean transformation with pyramid construct

The full length CDS of *AtNHX1* (GenBank accession number: EF596738; Locus: AT5G27150), as *EcoR*I and *BamH*I fragment, was cloned downstream to stress-inducible rd29A promoter in an intermediate vector pRT101. Following digestion with *Hind*III, the rd29A:*AtNHX1*::35STer fragment was inserted into binary plant vector pCAMBIA3300 that contained *bar* as plant selectable marker. The construct was mobilized into *Agrobacterium tumefaciens* strain EHA105 using Triparental mating prior to use in the plant transformation experiments.

Mungbean plants (cv. K-851) were transformed through *Agrobacterium*-mediated transformation of 4-d old cotyledonary node explants using *A. tumefaciens vir* helper strain EHA105, harboring pCAMBIA3300-rd29A::*AtNHX1*. Prior to inoculation in bacterial suspension for 30 min at 22°C with gentle shaking (90 rpm), the explants were pricked 3–4 times with a fine hypodermic needle (24G) at the regeneration site. The explants were then blotted dry on sterile filter paper and co-cultivated on filter paper disc moistened with liquid cocultivation medium LCM [MSB5 medium (Murashige and Skoog, 1962) containing 1 μM BAP, pH adjusted to 5.5] supplemented with 100 μM acetosyringone for 3 days at 22°C in dark condition. After cocultivation, the regenerating explants were selected on shoot induction and selection medium [MSB_5_ medium containing 5.0 μM benzylaminopurine (BAP), 1.5 mg/l phosphinothricin (PPT), and 500 mg/l cefotaxime], followed by subculturing of explants after 2 weeks on shoot elongation medium (MSB_5_ medium containing 1.0 μM BAP and 500 mg/l cefotaxime) devoid of PPT. After 4 weeks, the elongated shoots were excised and rooted in B_5_ medium (Gamborg et al., 1968) supplemented with 5 μM indolebutyric acid (IBA) and 500 mg/l cefotaxime. The putative transformed plants were established in soil: compost (1:1) in pots. The pots were covered with polythene bags in a transgenic greenhouse containment facility at 28 ± 2°C under 16/8 h photoperiod (fluence density of 50 μmol m^−2^ s^−1^) and 65–70% relative humidity. After 15 days, the plastic covers were gradually removed and the plants were transferred to soil in pots which were maintained for the production of the homozygous T_1_, T_2_, and T_3_ generations. One month-old plants were used for PCR screening and for gene expression evaluations. Positive transformants were used further for stress treatments.

### Molecular characterization of transgenic mungbean plants

The genomic DNA was isolated from young leaves by using DNASure Plant Mini Kit (Nucleopore, Genetix, India). Presence of transgenes in transformed plants (T_0_, T_1_, and T_2_) were confirmed by PCR using *bar* and *AtNHX1* specific primers (Tables S1, S2). Southern hybridization was carried out to confirm the transgenes integration and determine their copy number by digesting 50 μg of genomic DNA each from the WT and randomly selected independent T_2_ transgenic lines (AT_2_.1, BT_2_.5, CT_2_.3, DT_2_.2, and ET_2_.4) with *EcoR*I. After resolving the digested DNA on 0.8 % agarose gel, the gel was blotted onto positively charged Zeta-Probe membrane (Bio-Rad, Hercules, CA, USA). The blot was hybridized with the DIG-labeled 0.3 kb PCR product, corresponding to the coding region of *bar*. Southern hybridization was performed according to the manufacturer's instructions of the DIG Labeling and Detection kit (Roche Diagnostics, Mannheim, Germany). Four randomly selected T_0_ PCR-positive events (AT_2_.1, BT_2_.5, CT_2_.3, DT_2_.2, and ET_2_.4) were advanced up to T_2_ generations, and segregation studies was carried out to determine the inheritance of *AtNHX1* and *bar* by PCR as described earlier.

### PPT resistant assay

The PPT dose that impose bleaching and necrosis of leaves of 2-month old T_2_ plants was determined by detached leaf disc assay by floating leaf discs of 1 cm diameter with aqueous solution of PPT (1, 2, 3, and 4 mg/l) for a week (data not shown). T_2_ plants were also sprayed on both sides of leaves with aqueous solution of PPT (2 mg/l). The vegetative injury level of both transformed and control WT was recorded after 2 weeks of PPT spray assay. The leaf discs were also tested by chlorophenol red (CPR) assay to visually verify *bar* activity in 22 transgenic T_2_ plants representing six PCR positive T_0_ parents, as per the procedure described by Sonia et al. (2007).

### Salt stress tolerance assays

T_2_ lines of four independent transgenic events (AT_2_.1, CT_2_.3, DT_2_.2, and ET_2_.4) with abundant transcripts of *AtNHX1* and no apparent phenotypic changes, were chosen as representatives to evaluate salt tolerance by subjecting both the seedling and mature plant stage to salinity stress tests, and leaf senescence assay. Five-day old seedlings were treated with 150 mM NaCl for 2-d followed by recovery on Hogland's medium for 3-d. Similarly, 1-month old plants in pots were subjected to 150 mM NaCl for 1 week, by incremental increase of NaCl stress by 50 mM every 2 d. Subsequently, plants were treated with 200 mM NaCl (lethal dose) for 30 days based on prior determination of salt concentrations which imposed salt induced symptoms. To determine the relative increase in growth difference of the transgenics in salt relative to control conditions, we measured growth parameters (root and shoot length, fresh and dry weight of root and shoot) prior to, during and after the withdrawal of salt stress, and expressed as a percentage reduction in comparison with the respective unstressed control.

Mature leaves (from the top) from WT and T_2_ transgenic lines (45-d old) were harvested for leaf senescence assay (Sahoo et al., 2016). Leaves cut into leaf discs were floated in NaCl solution (100, 150, and 200 mM) for 4 days. Total chlorophyll content was determined by the procedure following the description in Arnon (1949). Briefly, leaf tissue (100 mg) were homogenized thoroughly in 80% acetone at 4°C. The homogenate was centrifuged at 3,000 rpm for 10 min in the dark. The absorbance of the supernatant was measured at 648 and 664 nm, and chlorophyll content was calculated per gram of fresh weight. The experiment was repeated three times. The chlorophyll amount was calculated using the formula:

$$\begin{array}{l} \text{Chl }\left(\text{a}+\text{b}\right)=\left(8.02\text{ x }{\text{A}}_{664}+20.2\;x\;A_{648}\right)V/W \end{array}$$

where, V is the volume of extract in ml and W is the weight of leaf tissue in g, Chlorophyll content was expressed in μg/g fresh weight.

### Physio-biochemical screening of transgenic plants

Leaves and roots of transgenic (AT_2_.1, CT_2_.3, DT_2_.2, and ET_2_.4) and WT plants were sampled, prior to, during and after the withdrawal of the salt stress for determination of leaf senescence, chlorophyll content, relative water content (RWC), electrolytic leakage, malonaldehyde (MDA), ascorbate, proline, and trehalose contents, chlorophyll fluorescence, stomatal conductivity, and transpiration rate.

RWC was determined according to Lv et al. (2009). Briefly, the leaf discs were floated, after recording the fresh weight, on deionized water for 5 h at 28°C, after which the turgid weight (TW) of the hydrated leaf discs was recorded. The leaf discs were then dried in a hot air oven at 80°C for 72 h and weighed until a consistent dry weight (DW) was obtained. RWC was calculated using the formula,

$$\begin{array}{l} \text{RWC }\left(\text{\%}\right)=\left(\text{TW}-\text{DW}\right)/\left(\text{FW}-\text{DW}\right)\times 100. \end{array}$$

Electrolyte leakage was estimated (Dionisio-Sese and Tobita, 1998) by measuring the electrical conductivity (ECa) of 25 leaf disks dipped in deionized water. Subsequently, the test tubes containing the leaf discs were incubated in water bath for 25 min. at 50–60°C, and the electrical conductivity (ECb) of the samples was determined. Finally, the samples were boiled at 100°C for 10 min, and then the electrical conductivity (ECc) was measured. The electrolytic leakage was calculated using the following formula:

$$\begin{array}{l} \text{Electrolyte leakage }\left(\%\right)=\left\{\left(ECb-ECa\right)\times 100\right\}/ECc \end{array}$$

Lipid peroxidation was estimated by measuring the malondialdehyde equivalents following the method (Heath and Packer, 1968). About 200 mg of fresh leaf tissue was homogenized with 2–3 ml of 0.25% TBA containing 10% TCA (trichloroacetic acid). The homogenate mixture was boiled in water bath at 95°C for 30 min, cooled to room temperature and centrifuged at 10,000 g for 10 min. Absorbance was measured at 532 nm and values corresponding to non-specific absorption at 600 nm were subtracted. The lipid peroxidation rate equivalents were calculated according to the molar extinction coefficient of MDA (155 mM^−1^ cm^−1^). The MDA content was calculated using the equation: MDA (μmolg^−1^FW) = (6.45 × O.D._532_ – (0.56 × O.D_450_).

Proline content was determined by extracting the samples in 3% sulfosalicylic acid and absorbance measured at 520 nm as described by Bates et al. (1973) and ascorbate content was measured as described by Panda et al. (2003). In addition, leaves and roots were harvested for Na^+^ and K^+^ detection respectively, using the method described by Wang et al. (2009).

Trehalose sugar content was estimated by following the method (Goddijn et al., 1997). Extracts from 300 mg of homogenized fresh leaf tissue were centrifuged at 3,220 × g for 10 min. and the supernatant were passed through ion-exchange columns to remove the charged compounds. After lyophilization, samples were dissolved in HPLC grade water and subjected to high performance ion exchange chromatography. Carbohydrates were eluted at a flow rate of 1 ml/min. at 1,400 psi with 100 mM NaOH for 30 min. Soluble sugar present in the extract were quantified by using authentic standard sugars (Sigma). The identification of trehalose in the extract was finally confirmed by incubation of samples with porcine kidney-derived trehalose enzyme (Sigma, USA).

The chlorophyll fluorescence (Fv/Fm) were measured using MINI-PAM-II, Photosynthesis Yield analyzer (Heinz Walz GmbH, Germany), and stomatal conductance and transpiration rate were measured by Leaf Porometer (DECAGON Devices, USA). Measurements were made on each plant in the five salinity treatments. The photosynthetic and chlorophyll fluorescence measurements were taken between 10:00 and 12:00 h when the ambient light intensity was 1,000 −1,200 μmol m^−2^ s^−1^.

The activity of superoxide dismutase (SOD), ascorbate peroxidase (APX), catalase (CAT), and glutathione reductase (GR) was evaluated as described earlier (Luck, 1974; Sahoo et al., 2016). Analysis of ROS accumulation in leaves of T_2_ transgenics (AT_2_.1, CT_2_.3, DT_2_.2, ET_2_.4, and CT_2_.9) and WT under normal and stress conditions was carried out by measuring hydrogen peroxide (H_2_O_2_) and superoxide radical (${\text{O}}_{2}^{-}$) as previously described by Ramel et al. (2009), and Rao and Davis (1999), respectively.

### Oxidative stress analysis

Methyl vialogen (MV) induced oxidative stress analysis was performed in WT and transgenic lines (AT_2_.1, CT_2_.3, DT_2_.2, ET_2_.4, and CT_2_.9) in T_2_ generation by leaf disc assay (Lee et al., 2007) with slight modifications. In brief, leaf discs (1 cm in diameter) from the third or fourth leaf from the top, of 8 week-old plants were floated on an aqueous solution of MV (0, 50, 100 μM) to generate superoxide radicals. They were incubated in dark at 25°C for 12 h to allow for MV diffusion. After pre-incubation, the leaf discs were illuminated (100 μmolm^−2^s^−1^) until use. The effect of MV on leaf discs was analyzed by monitoring their phenotypic changes and measuring the content of chlorophyll, proline, and MDA as described previously.

### Real-time RT-PCR analysis

To assess the effect of salt stress inducible expression of *AtNHX1* in transgenic mungbean plants, quantitative real-time PCR (qRT-PCR) assay was performed on four Southern positive plants (AT_2_.1, CT_2_.3, DT_2_.2, and ET_2_.4 in T_2_ generation) and the WT subjected to 200 mM salt stress. Leaves collected from the plants, after 2-d at seedling stage and 30-d at maturity stage of stress treatment were used for qRT-PCR analysis using the method as described in our previous studies (Sahoo et al., 2016). Three biological replicates and three technological replications were conducted. The primers used are listed in Tables S1, S2. Expression of mungbean β*-tubulin* gene served as internal control.

### Assessment of salinity tolerance in transgenics and evaluation of yield related traits

The growth parameters at both seedling and mature stages, and yield components of transgenic lines (AT_2_.1, CT_2_.3, DT_2_.2, ET_2_.4, and CT_2_.9) in T_2_ generation were evaluated under salt stress (200 mM), and compared to that of WT and control conditions. The growth parameters (plant height and number of branches) were checked on 0- and 45-d of salt stress. At harvest maturity, number of pods, number of seeds, 100-seed weight, and 10-seed length on a per plant basis were determined. Sodium and potassium ion concentrations in shoot and root of mature plants were determined at 0 and 48 h of salt stress. Transgenic as well as the WT and other control plants were washed in flowing tap water for 30 s and oven-dried for the measurement of ion content.

### Statistical analysis

All assays were biologically replicated at least three times. The data were evaluated with Statistical Package for the Social Sciences (SPSS 16.0) and Excel. The significant differences between mean values were determined by using Bonferroni analysis at *p* = 0.05.

## Results

### Generation and molecular characterization of transgenic mungbean plants

**Figure 1:**
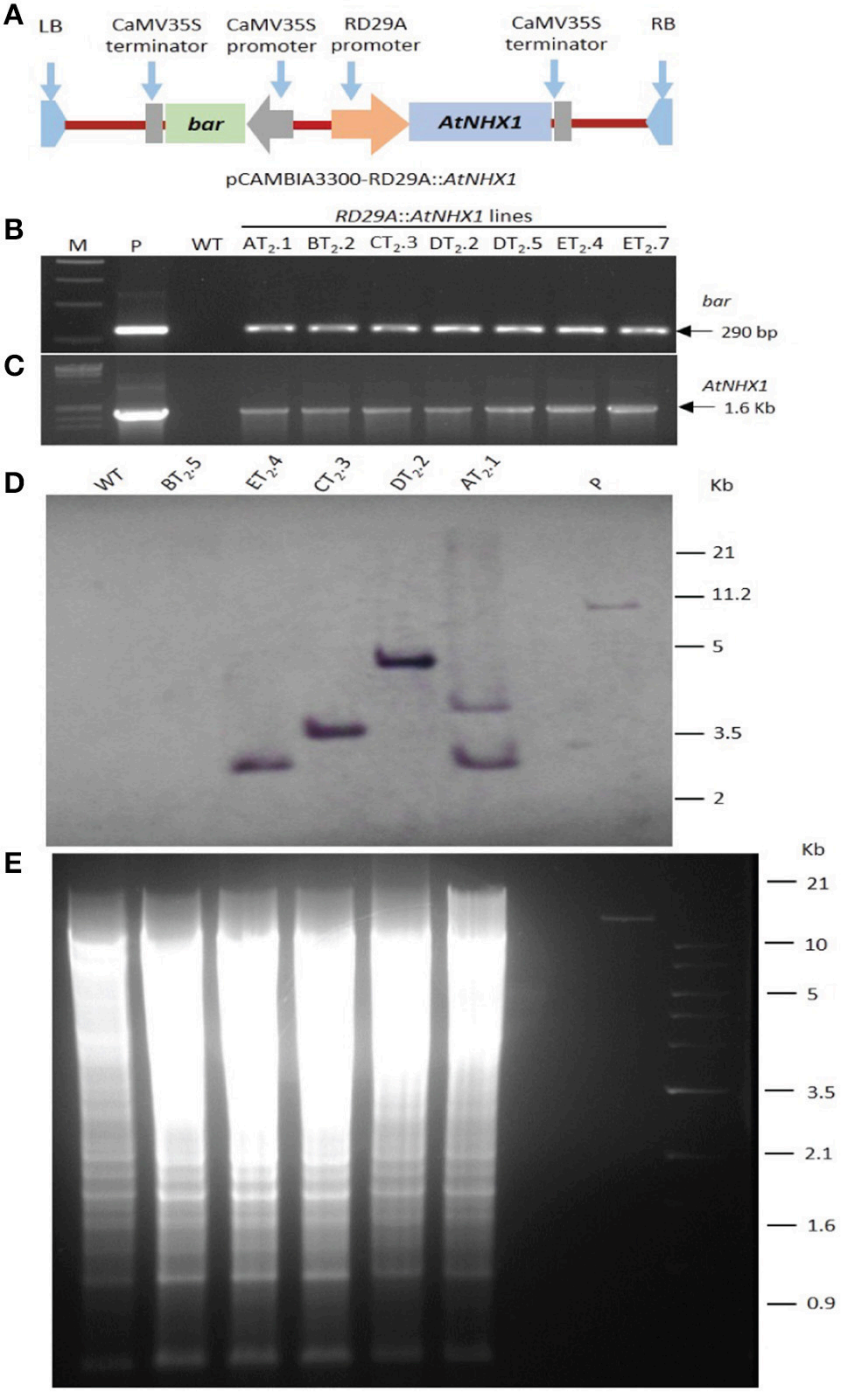
Molecular characterization of *AtNHX1-bar* mungbean lines. Schematic diagram of T-DNA region of the binary vector pCAMBIA3300-RD29A::AtNHX1 **(A)** PCR detection of 290 bp expected product with *ba*r specific primer **(B)** and 1.6 Kb expected product with *AtNHX1* specific primer **(C)**, Lanes M, 1 kb DNA marker; lane 2, pCAMBIA3300-RD29A::AtNHX1; lane WT, untransformed plant control; *RD29A*::*AtNHX1* lines, DNA from putative transformants; Southern blot of *Eco*RI digested genomic DNA of five independent T_2_ transgenic lines and WT **(D)**, hybridization using *bar* probe. Genomic DNA southern gel run along with DNA marker **(E)**.

Transgenic mungbean plants harboring both *bar* and *AtNHX1* were generated through *Agrobacterium-*mediated transformation (Figure 1A, Figures S1A–G, and Table S3). The expected size amplified products of 290 bp (Figure 1B) and 1.6 kb (Figure 1C) confirmed the presence of *bar* and *AtNHX1*, respectively in PPT resistant T_0_ transgenic mungbean lines. The segregation of *bar* and *AtNHX1* in four T_0_ transgenic lines followed up to T_2_ generations (AT_2_.1, CT_2_.3, DT_2_.2, and ET_2_.4) were confirmed by the detection of expected size amplified products. Three transgenic lines (CT_2_.3, DT_2_.2, ET_2_.4) showed a segregation ratio of 3:1, well supported by the fact that a single functional T-DNA locus detected in these lines in Southern hybridization with *bar* probe (data not shown). The observed segregation ratio of *AtNHX1* and *bar* revealed their inheritance in a Mendelian fashion (data not shown). A differential integration pattern of the transgene was observed in four out of five lines in T_2_ generation (AT_2_.1, BT_2_.5, CT_2_.3, DT_2_.2, and ET_2_.4). Three lines exhibited single copy (CT_2_.3, DT_2_.2, and ET_2_.4) insertion, AT_2_.1 had two copies while BT_2_.5 had no signal of T-DNA for the probed region in the genome (Figure 1D). The occurrence of hybridization signals greater than 1.2 kb revealed stable integration of the transgenes. No hybridization signal was observed in the WT.

### Herbicide tolerance of T_2_ homozygous transgenic lines

**Figure 2:**
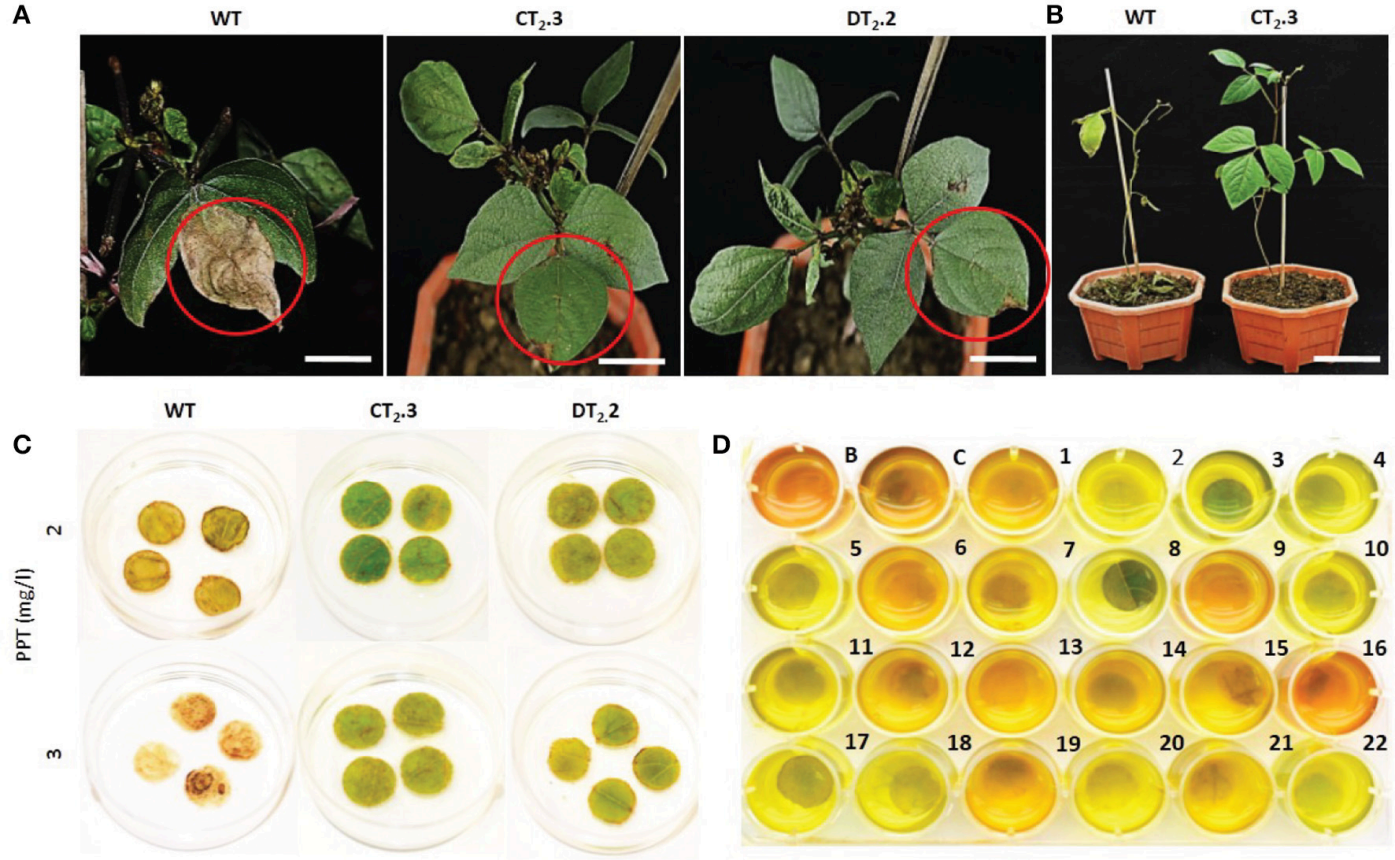
Herbicide tolerance analysis of *AtNHX1-bar* transgenic mungbean lines. Leaf necrosis test of T_2_ transgenic lines and WT; after 14-d of herbicide application over surface of solitary leaf **(A)**, on whole plants **(B)**, bar = 3.5 cm; PPT leaf disc necrosis test **(C);** chlorophenol red (CPR) assay on leaves of 22 T_2_ transgenic lines and WT **(D)**; B, blank; C, negative plant control.

Wild type mungbean plants were very sensitive to PPT painting, as the leaves exhibited complete necrosis at 2 mg L^−1^ (within a week in detached leaf disc assay and 2 weeks in leaf paint assay) while the PCR positive plants in T_2_ generation showed complete tolerance as the treated leaves stayed green without visible damage symptoms (Figures 2A–C). These results indicate that PCR based detection in combination with herbicide paint reliably eliminates the escapes. The color change of chlorophenol red assay solution is pH dependent as described by Treiu and Harrison (1996). The solution color at pH 6.0 and higher is red whereas the color is yellow at lower pH. The leaves tissue resistant to PPT cause acidification of the medium by metabolizing PPT leading to yellowing of the solution. The color of the medium in the chlorophenol red assay changed from red to yellow implying the bar gene activity in transformed leaf discs. Appearance of dark yellow color in medium in some wells indicated different expression levels of *bar* in those T_2_ lines (Figure 2D).

### Stress inducible expression of *AtNHX1* improved salt stress tolerance in transgenic mungbean

**Figure 3:**
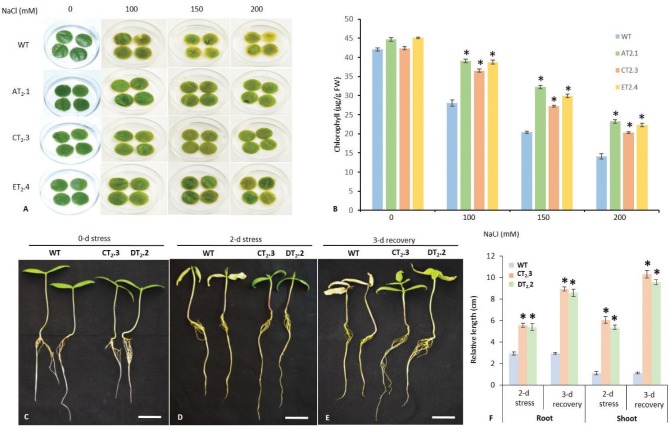
Salt stress analysis of *AtNHX1-bar* transgenic mungbean seedlings. Leaf disc assay for retardation of salt stress-promoted senescence in detached leaves of different T_2_ transgenic and WT plants indicating the tolerance at the cellular level to toxic levels of salt **(A)**. Discs floated on 100, 150, and 200 mM NaCl showed chlorophyll retention in transgenic lines, whereas WT plants showed bleaching of leaf discs compared with the untreated control (water); Total chlorophyll content of leaf discs of WT and transgenic plants exposed to salt stress (100, 150, and 200 mM NaCl) for 96 h AT_2_.1, CT_2_.3, ET_2_.4, T_2_ transgenic lines; WT, non-transgenic control **(B)**. Five-days-old different T_2_ transgenic and WT seedlings at 0-d **(C)**, seedlings after 2-d of salt stress (150 mM NaCl) for 2-d **(D)**, and after 3-d of recovery **(E)**, bar = 5; WT plants show severe chlorosis caused by Na^+^ toxicity, whereas the transgenic plants show normal phenotype. Comparison of relative root and shoot length of WT and different T_2_ transgenic lines after 2-d stress and 3-d recovery **(F)**. The data shows the mean ± S.E of three replicate samples. *Indicates significant differences from the WT at *P* < 0.05.

First, we examined the salt tolerance in leaf discs of transgenic plants grown in pots, as the degree of bleaching in leaf tissues was good indication of damage caused by salt stress in leaf senescence assay. Under control condition, no significant difference was observed between WT and transgenic plants. However, the leaf discs of WT plants started to turn yellow and exhibited excessive bleaching within a week under salt stress. In contrast, the leaves from transgenic lines continuously stayed green even after 15 days of 200 mM NaCl stress (Figure 3A) indicating higher tolerance to salt stress. Furthermore, leaf discs from transgenic plants showed significantly higher chlorophyll content, 1.68-, 1.76-, and 1.92-fold higher than WT under 100, 150, and 200 mM stress, respectively (Figure 3B).

**Figure 4:**
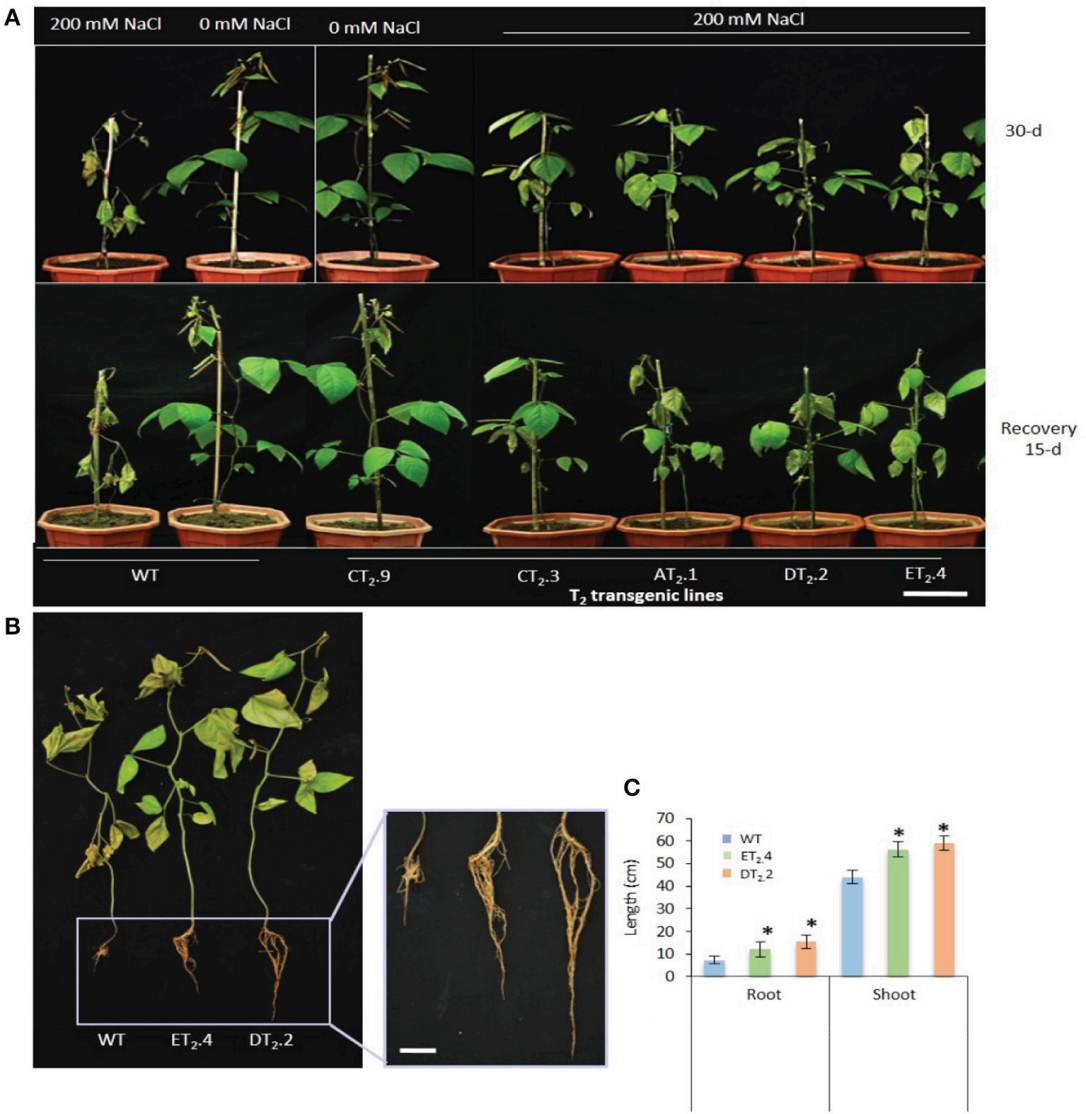
Response of *AtNHX1-bar* transgenic mungbean lines to salinity stress. **(A)** Five-week-old different T_2_ transgenic and WT plants subjected to salt stress (200 mM NaCl) for 30-d (top) and 15-d post recovery (bottom), bar = 10 cm. WT plants show severe chlorosis caused by Na^+^ toxicity, whereas the transgenic plants show normal phenotype; **(B)** Root phenotype analysis of different T_2_ transgenic and WT plants at 15-d post recovery, bar = 10 cm; **(C)** Comparison of root and shoot length of WT and different T_2_ transgenic lines grown on medium containing 200 mM NaCl. The data shows the mean ± S.E of three replicate samples. ^*^Indicates significant differences from the WT at *P* < 0.05. AT_2_.1, CT_2_.3, DT_2_.2, ET_2_.4, and CT_2_.9, T_2_ transgenic lines; WT, non-transgenic control.

Subsequently, we examined the effects of salt stress on the growth of T_2_ transgenic lines at seedling (T_2_.3 and DT_2_.2) (Figures 3C–F) and whole-plant stage (AT_2_.1, T_2_.3, DT_2_.2, ET_2_.4, and T_2_.9; Figure 4A) by subjecting them to 2 and 30-d of salt stress respectively. Under salt stress, transgenic lines exhibited no phenotypic difference from that in controlled condition (CT_2_.9) (Figures 3C–F, 4A). After irrigating with salt solutions, 2-d at seeding stage (Figure 3D) and for 30-d at maturity stage, with gradual incremental increase of NaCl dose to 200 mM, the WT plants developed severe growth inhibition, chlorosis, and wilting, and their growth ceased (Figure 4A). Furthermore, the WT plants showed significant decrease in shoot and root growth (Figures 3F, 4B,C). The WT plants showed no sign of recovery after 3-d at seedling stage (Figure 3E) and 15-d of withdrawal of salt stress at maturity stage (Figure 4A). However, transgenic plants stayed green and continued to grow normal at 200 mM NaCl and the symptoms of high salt stress were effectively alleviated, indicating that transgenic plants were more tolerant to high salt stress. The transgenic lines showed apparent advantages of growth under salt stress relative to control conditions as observed from their growth difference. The relative increase in growth indexes (shoot and root length) of transgenic lines under high salt stress (200 mM NaCl) were significantly higher (*P* < 0.05 and *P* < 0.01) than those under normal conditions (Figures 4B,C). These results implied that stress inducible overexpression of *AtNHX1* triggered a significant increase in growth and performance of transgenic plants under salt stress, and the resulted salt stress tolerant phenotype was associated with stress inducible expression of *AtNHX1*.

### Salt stress altered tissue Na^+^ and K^+^ content transgenic mungbean

**Figure 5:**
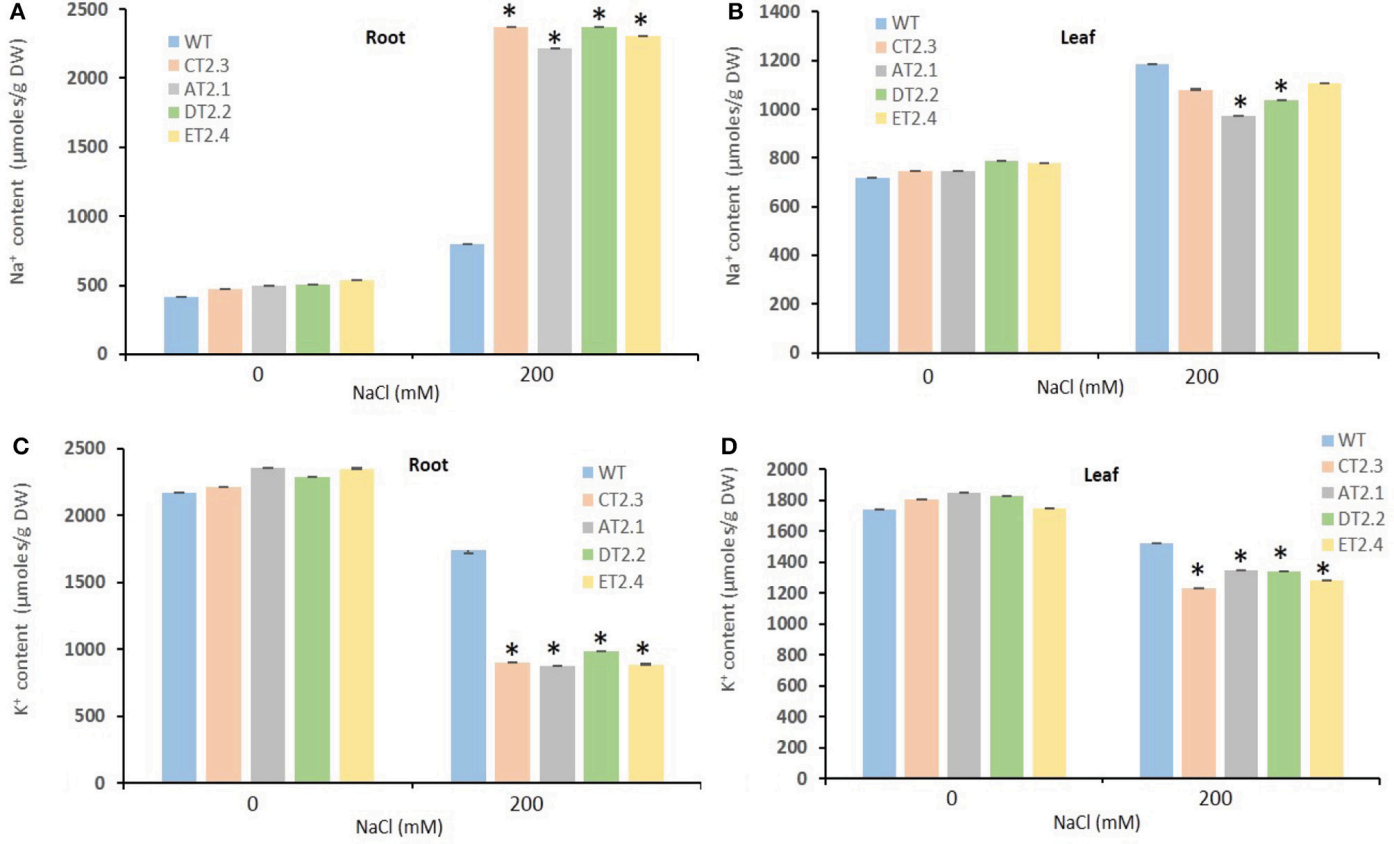
Intracellular Na^+^ and K^+^ ion content analysis of *AtNHX1-bar* transgenic mungbean lines under salt stress. Na^+^ content in roots **(A)** and shoots **(B)**, K^+^ content in roots **(C)** and shoots **(D)** of T_2_ transgenic and WT plants subjected to salt stress (200 mM NaCl) for 30-d. The data shows the mean ± S.E of three replicate samples. ^*^Indicates significant differences from the WT at *P* < 0.05. CT_2_.3, ET_2_.4, DT_2_.2, and AT_2_.1, T_2_ transgenic lines; WT, non-transgenic controls.

To investigate if the expression of *AtNHX1* altered the accumulation of cations (Na^+^ and K^+^) under salt stress conditions, the concentrations of cations were determined in the leaves and roots of the transgenic (AT_2_.1, CT_2_.3, DT_2_.2, and ET_2_.4) in T_2_ generation and WT plants. Under normal physiological condition, there was no significant difference in accumulation of Na^+^ and K^+^ in transgenic and WT plants (Figures 5A–D). On the other hand, salt stress (200 mM) resulted in enhanced accumulation of Na^+^ in transgenic and WT plants, in both leaves and roots. However, in transgenic plants (CT_2_.3 and DT_2_.2), the Na^+^ concentrations were significantly higher in roots and on the contrary lower in leaves, in comparison to WT plants (Figures 5A,B). The Na^+^ content in roots of T_2_ transgenic lines showed 2.95 (CT_2_.3)- and 2.76 (DT_2_.2)-fold higher accumulation than WT roots, under 200 mM salt stress (Figures 5A,B). These results clearly demonstrated that stress inducible expression of the Arabidopsis vacuolar antiporter elevated the accumulation of Na^+^ in roots, presumably in vacuoles, and the enhanced activity of vacuolar antiporters in roots possibly prevented the entry of Na^+^ into shoots. Lower Na^+^ content in leaves under high salt stress was evident from the absence of any salt induced damage in transgenic plants whereas WT plants demonstrated severe phenotype under similar conditions.

Salt stress induced a change (or reduction) in K^+^ uptake in both WT and transgenic plants, and the K^+^ content in leaves and roots of transgenic plants were significantly lower than that of WT plants (Figures 5C,D). High salt stress condition might have resulted in competition between Na^+^ and K^+^ for binding to same carriers resulting in impaired K^+^ uptake. Nevertheless, the growth of the transgenic plants remained unaffected by high salinity, indicating that K^+^ nutrition was not compromised. Consequently, the Na^+^/K^+^ ratio in leaves and roots was lower in transgenic lines than that in WT plants.

### Transgenic mungbean expressing *AtNHX1* are tolerant to salt induced osmotic stress

**Figure 6:**
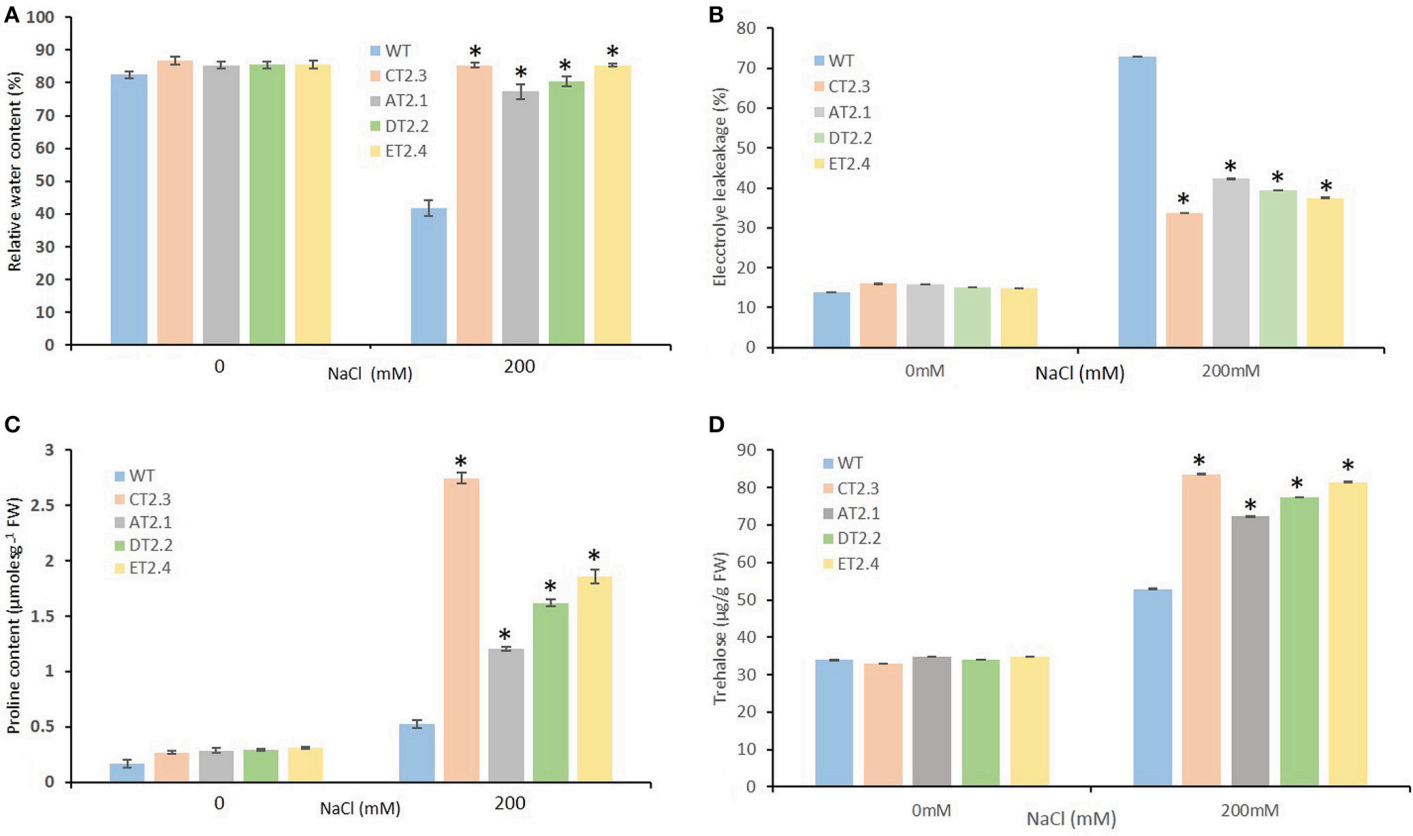
Osmotic stress response of *AtNHX1-bar* transgenic and wild type mungbean plants under salt stress. **(A)** Relative water content (percentage) in leaves of control untransformed (WT) and transformed T_2_ lines exposed to 200 mM NaCl stress for 30-d; **(B)** percentage electrolyte leakage from mungbean leaf tissue; **(C)** Changes in the level of proline accumulation; **(D)** Changes in the level of trehalose accumulation. The data shows the mean ± S.E of three replicate samples. ^*^Indicates significant differences from the WT at *P* < 0.05.

For the survival of plants under stress conditions, securing water balance is essential. Therefore, we examined the leaf RWCs in transgenic and WT plants during salt stress. With the imposition of high salt stress (200 mM NaCl), the leaf RWC declined in WT plants by 2-fold, on the contrary the RWC remained unaffected in transgenic lines (Figure 6A). The transgenic lines (T_2_ lines, AT_2_.1, CT_2_.3, DT_2_.2, and ET_2_.4) in T_2_ generation demonstrated approximately 2-fold higher potential to maintain leaf tissue water content than WT plants under high salt stress (Figure 6A). Our results indicated that transgenic plants overexpressing *AtNHX1* retained significantly higher water in their leaves under salt stress.

Disruption of the membrane integrity induced by high salinity stress can be estimated by measuring the leakage of cytoplasmic solutes from leaf. No difference was observed in electrolyte leakage between WT and transgenic mungbean plants under normal condition, while under high salinity stress (200 mM) condition, Electrolytic leakage (EL) in transgenic (T_2_ lines, AT_2_.1, CT_2_.3, DT_2_.2, and ET_2_.4) demonstrated a marked decrease (1.8- to 2.0-fold) (Figure 6B). Our results indicated that transgenic plants overexpressing *AtNHX1* retained significantly higher water content and electrolytes in their leaves under salt stress.

Accumulation of compatible osmolytes like proline in cell is known to protect against abiotic stresses through osmoprotection or osmoregulation. To evaluate if *AtNHX1* overexpression induced proline accumulation, the proline content was measured in the T_2_ transgenic and WT plants. No significant differences in the proline content were found between the WT and transgenic plants in normal condition. With the imposition of salt stress, the proline content increased in both WT and transgenic plants. However, transgenic plants recorded a significantly higher proline accumulation than WT plants, indicating that proline was involved in salt tolerance through efficient maintenance of higher cellular water balance (Figure 6C). The increase in proline content in transgenic plants (T_2_ lines, CT_2_.3, ET_2_.4, DT_2_.2, and AT_2_.1) was in the order of 5.18-, 3.50-, 3.11-, and 2.27-fold higher than those in WT plants under 200 mM NaCl stress, respectively (Figure 6C).

Significant amount of trehalose sugar were detected in transgenic mungbean plants under salinity stress. The transgenic plants grown under control conditions exhibited trehalose levels comparable with the WT plants. After 200 mM NaCl stress, the transgenic lines showed 1.4- to 1.8-times higher shoot trehalose levels compared with WT plants. The difference in trehalose levels between transgenic and WT plants clearly correlates with increased tolerance to salinity stress (Figure 6D).

### Transgenic mungbean expressing *AtNHX1* show improved tolerance to both salt- and MV-induced oxidative stress

**Figure 7:**
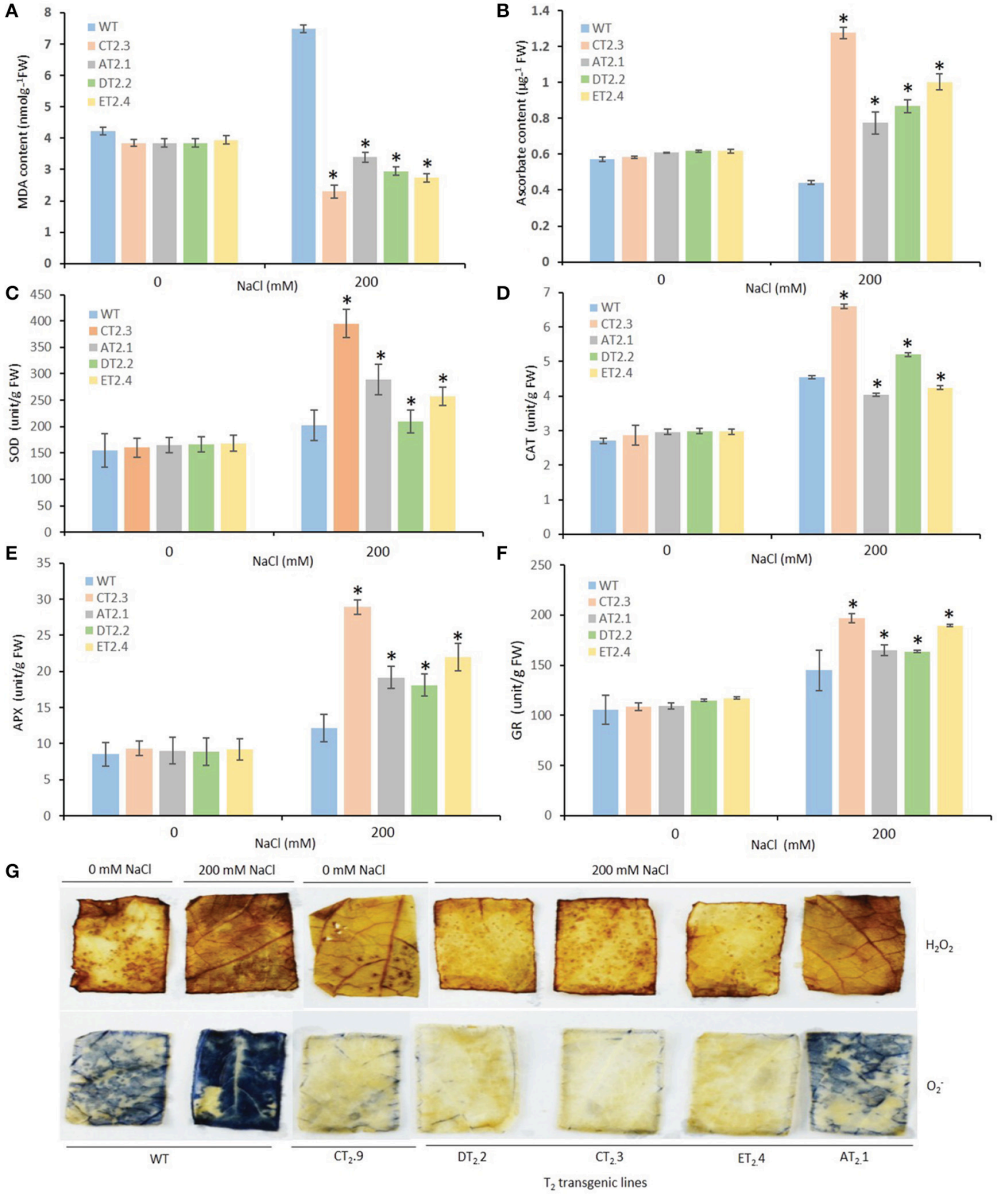
Antioxidative enzyme response of *AtNHX1-bar* transgenic mungbean lines under salt stress. **(A)** Levels of lipid peroxidation expressed in terms of MDA content; **(B)** Ascorbate content; **(C)** Changes in superoxide dismutase enzyme activity (SOD); **(D)** Changes in catalase enzyme activity (CAT); **(E)** Changes in ascorbate peroxidase enzyme activity (APX); **(F)** Changes in glutathione reductase enzyme activity (GR) activity in leaves of control untransformed (WT) and transformed T_2_ lines exposed to 200 mM NaCl stress for 30-d; **(G)** Histochemical analysis of oxygen radical and hydrogen peroxide in leaves of control untransformed (WT) and transformed T_2_ transgenic lines exposed to 200 mM NaCl stress for 30-d. The data shows the mean ± S.E of three replicate samples. ^*^Indicates significant differences from the WT at *P* < 0.05.

Salt stress exerts oxidative stress in plant cells in addition to ion toxicity and osmotic stress. ROS-scavenging is a well-known phenomenon, which play important role in salt tolerance. Therefore, we determined in transgenic plants, the possible role of *AtNHX1* overexpression in imparting elevated tolerance to oxidative stress. Lipid hydroperoxidation and H_2_O_2_ generation are effective indicators of cellular oxidative damage. We examined the changes of lipid hydroperoxide production rates by determining MDA content in the leaves of WT and transgenic plants. At high-salinity (200 mM NaCl) condition, MDA content significantly increased in the leaves of WT plants, whereas the transgenic plants (T_2_ lines, AT_2_.1, CT_2_.3, DT_2_.2, and ET_2_.4) demonstrated a marked decrease (0.39- to 1.73-fold) in MDA content (Figure 7A). The lower MDA content in transgenic plants than that of WT indicated the lower membrane injury of transgenic plants under high salt stress.

Among the antioxidants, ascorbate plays a central role in defense against oxidative stress. Protective functions provided by ascorbate and related antioxidant enzymes against photooxidative stress in chloroplasts have also been reported. No significant differences in the ascorbate content were found between the normal and salt stress (200 mM NaCl) in WT plants. However, the ascorbate contents of transgenic plants (T_2_ lines, AT_2_.1, CT_2_.3, DT_2_.2, and ET_2_.4) were approximately 1.6- to 3.0-fold higher in salt stress condition (Figure 7B). The increase in the reduced ascorbate content may have role in increase in redox state in the transgenic lines. Activity of antioxidant enzymes (SOD, CAT, APX, and GR) was higher in both WT and transgenic plants (T_2_ lines, AT_2_.1, CT_2_.3, DT_2_.2, and ET_2_.4) up on exposure to 200 mM NaCl (Figures 7C–F). Among the antioxidant enzymes, the maximum increase was noticed in APX followed by SOD, CAT, and GR in both WT and transgenic lines (Figures 7C–F). The transgenic lines recorded an increase of 2.5-fold of APX, 2.3-fold of SOD, 2.2-fold of catalase, and 2.1-fold of GR in comparison to WT plants, under salt stress condition. Enhanced activity of antioxidant enzymes may have played a role in detoxification of H_2_O_2_ formed during photosynthesis and photorespiration.

We also performed H_2_O_2_ detection using DAB staining method. Formation of brown precipitate against H_2_O_2_ indicates the polymerization of DAB, was detected in both WT and transgenic lines. However, complete bleaching at the end of the leaf segments was observed in salt-stressed WT plants, whereas those from transgenic plants (T_2_ lines, AT_2_.1, CT_2_.3, DT_2_.2, and ET_2_.4) showed significant tolerance to H_2_O_2_-induced damage (Figure 7G). The NBT staining for O^2−^ detection after salt stress treatment revealed accumulation of O^2−^ in the form of intense blue color in WT whereas negligible staining appeared in transgenic lines indicating lower O^2−^ generation and lesser oxidative damage in transgenic plants.

**Figure 8:**
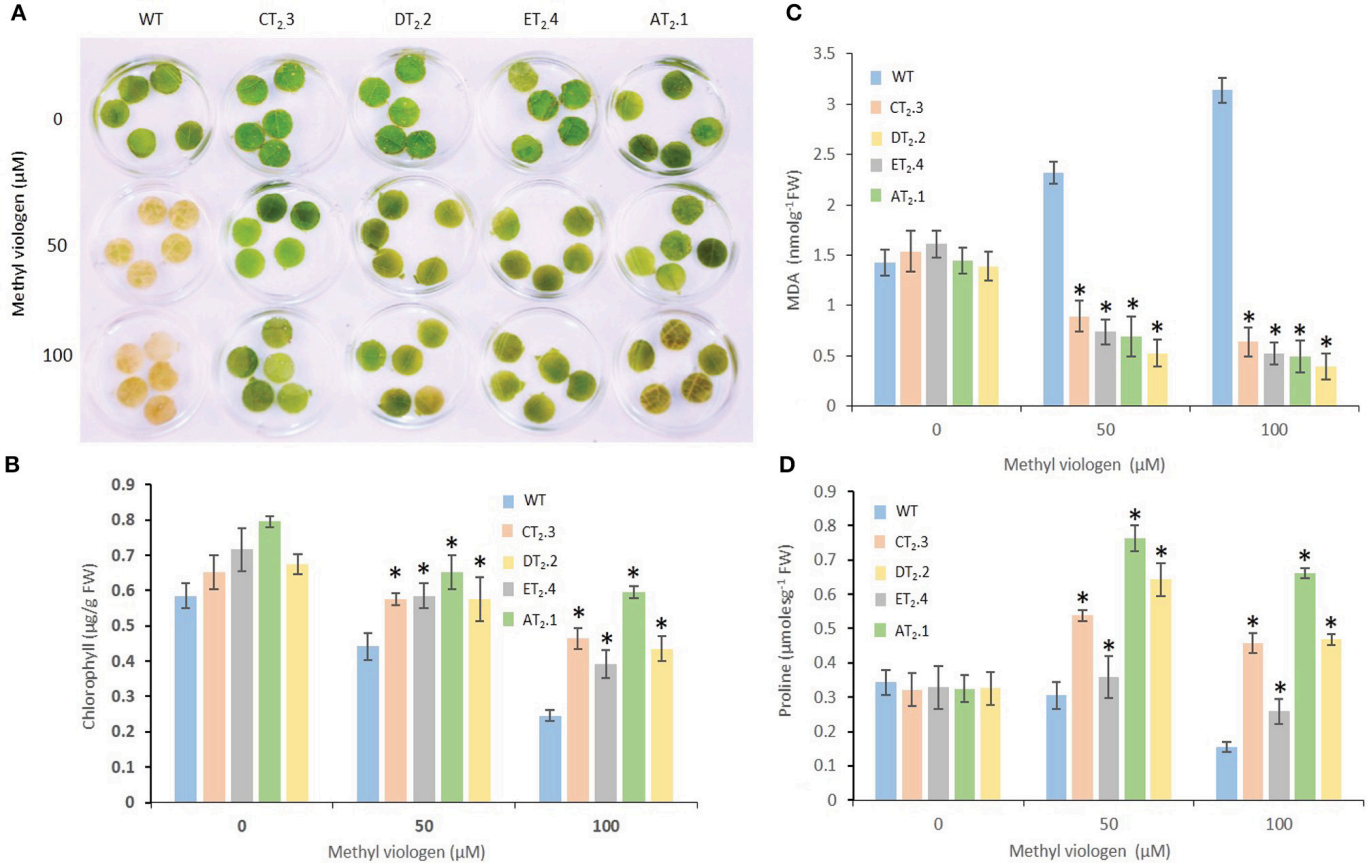
Oxidative stress tolerance test of *AtNHX1-bar* transgenic mungbean lines under salt- and MV-induced stress. **(A)** Leaf disc assay for oxidative stress tolerance in WT and T_2_ transgenic mungbean lines incubated at different concentrations of methyl viologen (MV) for 12-h; **(B)** Quantification of chlorophyll content from the leaf disk assay. Leaf disc floated in water served as control; **(C)** Levels of lipid peroxidation expressed in terms of MDA content; and **(D**) Changes in the level of proline accumulation. The data shows the mean ± S.E of three replicate samples. ^*^Indicates significant differences from the WT at *P* < 0.05.

To evaluate direct impact of oxidative stress on *AtNHX1* overexpressing transgenic mungbean plants, the leaf discs were treated with MV, a superoxide-generating herbicide. Severe necrosis was observed in the leaf discs of WT plants treated with MV while partial necrosis was detected at the peripheries of the leaf discs of the transgenic plants (T_2_ lines, AT_2._1, CT_2._3, DT_2._2, and ET_2._4) indicating reduction in membrane damage due to the lower conductivity of the MV solution (Figure 8A). The photosynthetic efficiency of the leaf discs after MV treatment was evaluated in order to determine the extent of damage induced by MV treatment on the photosynthetic apparatus. No significant differences in the photosynthetic efficiency in leaf discs of the transgenic plants were noted in the absence of MV treatment. However, on exposure to MV stress, higher chlorophyll retention (Figure 8B), lower MDA content (Figure 8C), and proline content (Figure 8D) and were detected in transgenic plants suggesting only negligible effect on photosynthetic efficiency. On the contrary, photosynthetic efficiency was significantly reduced and extent of oxidative stress effect was severe in the WT plants (Figures 8A,D). These results suggest that *AtNHX1* overexpression in mungbean plants improve the tolerance to oxidative stress.

**Figure 9:**
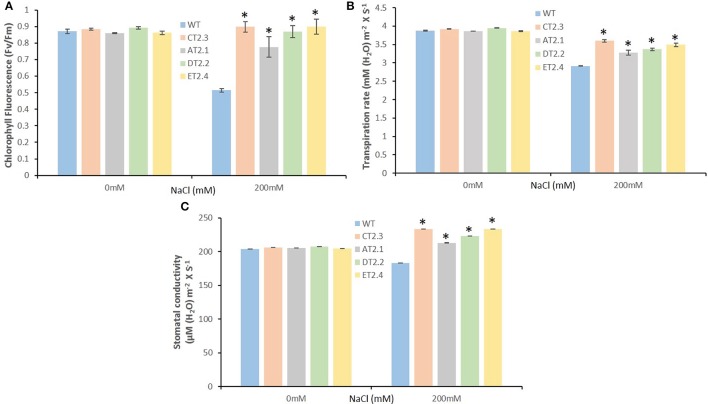
Measurement of photosynthetic efficiency of *AtNHX1-bar* transgenic mungbean lines under salt stress. **(A)** PSII photosynthetic efficiency (Fv/Fm) in the leaves of mungbean plants; **(B)** transpiration rate; **(C)** stomatal conductance. The data shows the mean ± S.E of three replicate samples. ^*^Indicates significant differences from the WT at *P* < 0.05.

To investigate the changes in PSII level, photoinhibition, utilization, and dissipation of excess excitation energy in salt-stressed mungbean transgenics, measurements of various fluorescence parameters related to PSII photochemistry were analyzed for both the control and salt stressed plants. The maximum efficiency of PSII (Fv/Fm) was measured to see whether the sensitivity of the plants to photoinhibition was increased. We found significant differences from 1.4- to 1.8-fold in the Fv/Fm between the control and salt treated mungbean plants (Figure 9A).

The transpiration rate and stomata conductivity was in accordance with the stomatal conductance. The rate of transpiration was not decreased significantly at higher salinity. At lower salt concentration (250 mM), the transpiration rate was identical to control value, but at high salinity, the transpiration rate was found significantly higher from 0.92- to 1.1-fold in accordance with higher stomatal conductivity from 1.1- to 1.4-fold in compare to the WT mungbean plants (Figures 9B,C).

### Expression analysis transgenic mungbean under salt stress

**Figure 10:**
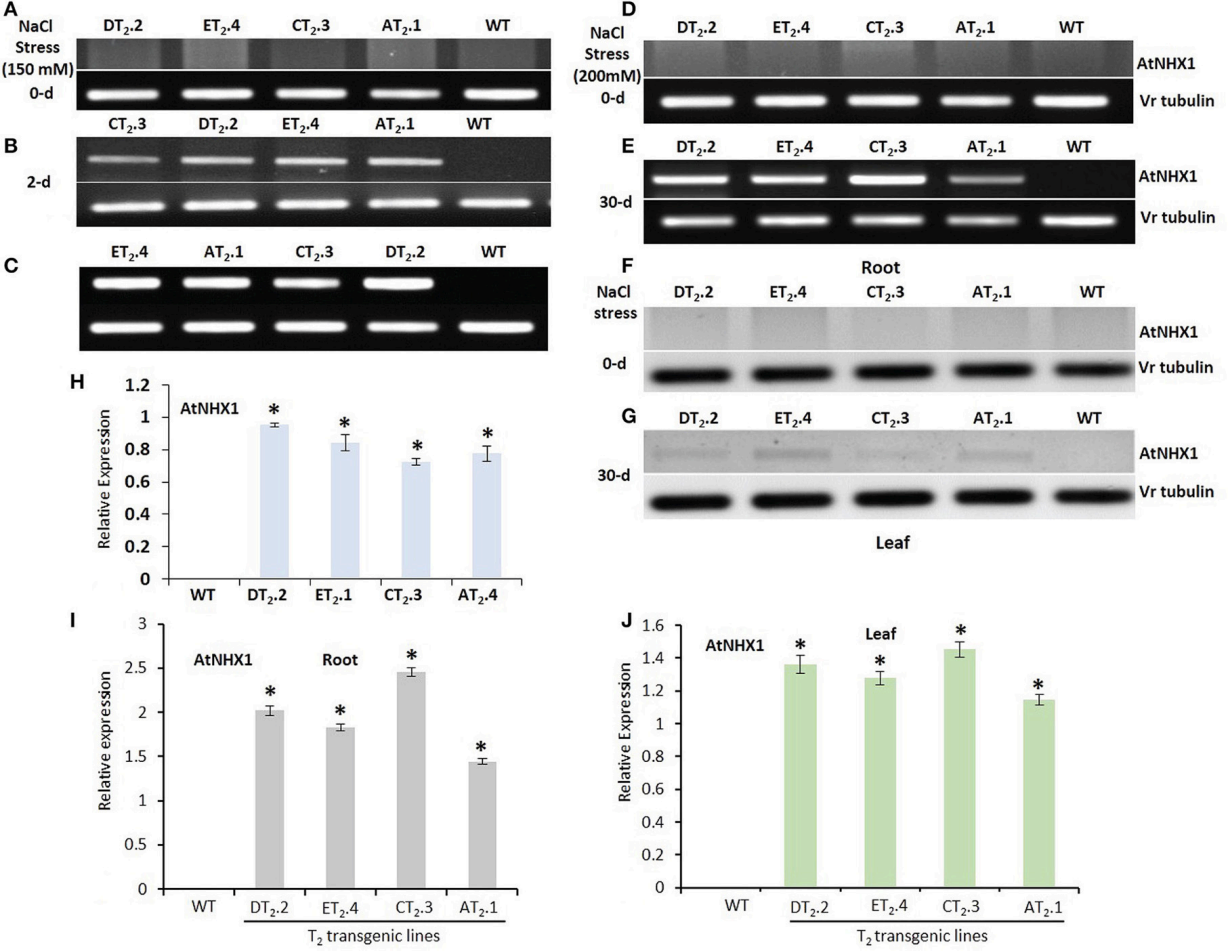
Relative transgene expression level of *AtNHX1* and *bar* in *AtNHX1-bar* transgenic mungbean lines. RT-PCR analysis of *AtNHX1* expression in WT and T_2_ transgenic mungbean seedlings at 0-d **(A)** and after 2-d of salt stress **(B)**, RT-PCR analysis of *bar* expression **(C)**; RT-PCR analysis of *AtNHX1* expression in WT and T_2_ transgenic mungbean lines in roots after 0-d **(D)** and 30-d of 200 mM NaCl stress **(E)**, and in leaves after 0-d **(F)** and 30-d of 200 mM NaCl stress **(G)**; Real time qRT-PCR analysis of level of *AtNHX1* expression in T_2_ transgenic mungbean seedlings **(H)**; in roots **(I)** and in leaves **(J)** of T_2_ transgenic mungbean mature plants and WT; Vr-tubulin gene served as internal control. The data shows the mean ± S.E of three replicate samples. ^*^Indicates significant differences from the WT at *P* < 0.05.

The expression pattern of *AtNHX1* under stress inducible promoter rd29A was monitored in transgenic lines by qRT-PCR at seedling stage of T_2_ lines (CT_2_.3 and DT_2_.2) on shoots, and at maturity stage on both root and shoot (T_2_ lines, AT_2_.1, CT_2_.3, DT_2_.2, and ET_2_.4) and WT plants exposed to high salt stress (150 mM NaCl at seedling stage and 200 mM NaCl at maturity stage) and normal condition. Prior to qRT-PCR, the samples were subjected to semi-quantitative RT-PCR. The results of RT-PCR analysis indicated that *AtNHX1* and *bar* effectively expressed in all the transgenic plants (Figures 10A–G). The mungbean β*-tubulin* was used as internal control to normalize the expression of all genes. No transgene expression was observed in the WT. The salt stress dramatically induced the *AtNHX1* expression both in roots and leaves, as revealed by qRT-PCR. However, *AtNHX1* transcript accumulation was 1.5-fold higher in leaves of transgenics seedlings after 2-d of exposure to 150 mM NaCl, 2.5-, and 1.4-fold higher in root and shoot of mature plants after 200 mM NaCl treatment (Figures 10H–J). Among the transgenic lines, CT_2_.3 exhibited the highest expression, followed by DT_2_.2, ET_2_.4, and AT_2_.1 (Figures 10I,J).

### Evaluation of yield related traits

The transgenic lines grew well, flowered normally, and set pods and viable seeds even under 200 mM NaCl salt stress (Table S4). Although no significant difference was observed among different transgenic lines for plant height, branch number, pod number, seed number, seed weight per plant, and 10-seed-weight and length under 200 mM NaCl stress conditions, however, the WT plants showed significant reduction in these yield related traits under similar growth condition (Table S4). The WT plants displayed severe chlorosis, withered, and eventually died after 4 weeks of salt stress.

## Discussion

Salinity is a major abiotic stress that imposes severe detrimental effects on plant growth and causes significant reduction in crop productivity. Improving salt tolerance of crops is the most practical way to ensure crop protection to high soil salinity (Flowers, 2004; Bartels and Sunkar, 2005; Rozema and Flowers, 2008; Yang et al., 2010). Genetic engineering provides tools for imparting enhanced salt tolerance in crop plants. Plant tolerance to salt stress is a multigenic trait that involves various physiological and biochemical responses and concerted actions of several stress associated genes (Bartels and Sunkar, 2005). Among principal mechanisms, maintenance of ion homeostasis by ion uptake and compartmentalization is well known for normal plant growth during salt stress (Niu et al., 1995; Serrano et al., 1999; Hasegawa, 2013). In spite of the complexity of salt tolerance, introducing one single key gene, involved in stress tolerance has successfully enhanced salt tolerance in transgenic plants. Bassil et al. (2011) reported NHX functioning at the tonoplast affects plant growth and development by controlling cell expansion in vegetative tissues and male reproductive organs, and normal flower development and maintaining K^+^ homeostasis in the vacuole, especially in rapidly expanding cells of flowers and male reproductive organs. Their work clearly indicated how the protons pumped into the central vacuole in turn assisted in accumulation of vital cations for driving cell growth. Previous studies have demonstrated that overexpression of a single gene, vacuolar Na^+^/H^+^ antiporter gene associated with compartmentalization of Na^+^ in vacuoles, has been effective in conferring enhanced salt tolerance in various crop species, including grain legumes (Li et al., 2011; Mishra et al., 2014; Chen et al., 2015; Zhang et al., 2015; Wang et al., 2016; Huang et al., 2017). However, manifestation of salt tolerance through overexpression of vacuolar NHX must be mediated by a complex process involving other functional proteins. Studies on regulation of *BvNHX1* of salt-tolerant sugar beet plant in Arabidopsis and its expression patterns shown that the 5′ UTR of *BvNHX1*, including its intron, are not modulating the activity of the promoter. Mutation in *cis*-acting elements in its promoter revealed that MYB transcription factor(s) are involved in the activation of the expression of *BvNHX1* upon exposure to salt and water stresses (Adler et al., 2010). Salinity severely limits growth and yield of mungbean (Sehrawat et al., 2013). We recently reported constitutive expression of *AtNHX1* conferring enhanced salt tolerance to mungbean (Sahoo et al., 2016). However, growth and yield penalties can often be imminent in absence of stress or low stress environments, when the candidate genes are constitutively expressed (Roy et al., 2014).

Apart from the salinity stress, weed competitions are also one of the major problems for crop productivity. In mungbean, the yield loss due to weeds is as high as 65.4–79.0% (Shuaib, 2001; Dungarwal et al., 2003). Besides inflicting yield loss, weeds create competition for nutrients, space, and water and reduce the quality of produce (Arif et al., 2006). Mungbean is sown as broadcast, and therefore, it is difficult to weed them. The bar gene has been successfully used to develop transgenic plants with herbicide resistance (Fang et al., 2004; Manickavasagam et al., 2004; Sonia et al., 2007). Therefore, we decided to pyramid both salinity and herbicide tolerance, through stress inducible expression of *AtNHX1* and constitutive expression of *bar*.

In our work, T_2_ generation transgenic mungbean plants co-overexpressing *AtNHX1* and *bar* exhibited enhanced salt and herbicide tolerance. T_2_ seedlings and plants of *AtNHX1-bar* transgenics outperformed WT plants as they were able to survive, produced flowers and set pods with viable seeds under high salinity stress. These observations implicate that introduced traits in transgenic mungbean plants are functionally active and stable over generations. In addition to salinity stress, phosphinothricin spray on different transgenic T_2_ lines and WT plants revealed that the transgenic plants showed remarkably lesser injury and better survived, whereas WT plants succumbed to herbicide injury, withered, and finally died.

Under high salt stress, transgenic plants accumulated significantly higher Na^+^ in the roots (AT_2_.1, CT_2_.3, DT_2_.2, and ET2.4) and lower Na^+^ in the leaves (AT_2_.1 and DT_2_.2) compared to the WT, but showed less injury than WT. These results clearly demonstrated that stress inducible expression of the Arabidopsis vacuolar antiporter elevated the accumulation of Na^+^ in roots, presumably in vacuoles, and the enhanced activity of vacuolar antiporters in roots possibly prevented the entry of Na^+^ into shoots. Lower Na^+^ content in leaves (AT_2_.1 and DT_2_.2) under high salt stress was evident from the absence of any salt induced damage in transgenic plants whereas WT plants demonstrated severe phenotype under similar conditions. Futhermore, our results suggest that there may be increased Na^+^ compartmentation in vacuoles of transgenic mungbean roots resulting from enhanced tonoplast Na^+^/H^+^ exchange efficiency as a consequence of both improved expression and activity of the Na^+^/H^+^ transporters (Gaxiola et al., 2001, 2007; Leidi et al., 2010; Bao et al., 2014; Yang et al., 2015). This may have contributed to alleviating the toxicity of excess Na^+^ in the cytosol, maintaining intracellular K^+^/Na^+^ homeostasis, and enhancing vacuolar osmoregulatory capacity in roots (Apse et al., 1999; Blumwald, 2000; Gaxiola et al., 2007; Barragán et al., 2012; Flowers et al., 2015; Volkov, 2015; Yuan et al., 2015). Increased Na^+^ content in roots of transgenic mungbean lines as compared to WT in our observations is in accordance to that of Arabidopsis NHX double mutant showing increased Na^+^ content as compared to WT, at 100 mM NaCl (Barragán et al., 2012).

In transgenic mungbean plants, salt-tolerant phenotype was associated with lower Na^+^ content in leaves (AT_2_.1 and DT_2_.2) accompanied with a higher Na^+^ accumulation in the roots in comparison to WT plants, under salt stress. Overexpression of *AtNHX1* have promoted the active accumulation of Na^+^ in roots and the increased efficiency of transgenic root system might have limited the transport of ions to shoots thereby altering its distribution to leaves (Munns et al., 2006; Munns and Tester, 2008; Gill et al., 2013).

In accordance with normal plant phenotype of transgenic plants under high salt stress, results of our work indicate lower Na^+^ in leaves have benefited transgenic plants to withstand high salt stress. Our results are consistent with our previous observation of lower Na^+^ in leaves of salt tolerant cowpea transgenics, overexpressing *VrNHX1* (Mishra et al., 2014). Similar phenomena of lower Na^+^ in leaves transgenic plants overexpressing *NHXs* genes was also reported in rice (Islam et al., 2010), soybean (Li et al., 2010). In the transgenic plants examined, the salt-tolerant phenotype was associated with a lower K^+^ content in leaves and roots relative to WT plants. However, the impaired K^+^ uptake in transgenic plants, possibly due to membrane depolarization and repolarization across the plasma membrane (Shabala et al., 2015) and competition between Na^+^ and K^+^ for binding to same carriers (Anschütz et al., 2014), had not affected plant growth performance, physiological capacity and yield under high salt stress implying that K^+^ nutrition was not compromised.

Excess Na^+^ is known to cause an imbalance in cellular ions, inducing the generation of ROS, which consequently triggers chlorophyll degradation and membrane lipid peroxidation (Yasar et al., 2006). The adverse effect of salt stress directly affects the photosynthesis efficiency, stomatal conductivity, and transpiration rate which leads to reduction of the overall plant growth. The transgenic mungbean plants were found able to maintain higher chlorophyll fluorescence ratio (Fv/Fm), higher transpiration rate and higher stomatal conductivity under 200 mM NaCl stress in comparison to WT plants, which indicates better photosynthetic efficiency under high salt stress conditions (Maxwell and Johnson, 2000; Yusuf et al., 2010). *AtNHX1-bar* overexpressing transgenic plants showed lesser damage to photosynthetic apparatus, maintaining the normal growth and yield while the WT experienced severe damage of its photosynthesis machinery under 200 mM NaCl stress. The considerable protection of photosystem clearly indicated the ability of transgenic plants to ameliorate salt stress effects on photosynthesis.

The accumulation of high levels of endogenous proline and trehalose in *AtNHX1-bar* overexpressing transgenic plants, under high salt stress indicated its possible involvement in cellular protection and restoring water uptake through its osmolyte properties (Prasad et al., 1980; Kandpal and Rao, 1985). During salinity stress, the cellular osmotic potential against the osmotic gradient is eventually restored through accumulation of compatible solutes inside the cell thereby maintaining water uptake into roots (Li et al., 2014).

High salinity imposes, in addition to Na^+^ toxicity and osmotic stress, a secondary oxidative stress resulted due to the generation of ROS (Hasegawa et al., 2000). ROS accumulation during salinity stress affects the stability and integrity of membranes and cellular structures (Apel and Hirt, 2004; Gill and Tuteja, 2010; Gill et al., 2012, 2015; Das and Roychoudhury, 2014). *AtNHX1-bar* transgenic plants show accumulation of less H_2_O_2_ and O^2−^, significantly less lipid peroxidation, increased activities of antioxidant enzymes, higher accumulation of ascorbate and negligible leaf senescence indicating increased tolerance to oxidative stress.

*AtNHX1-bar* overexpressing transgenic plants showed up-regulation of *AtNHX1* upon salt stress treatment in both roots and leaves as opposed to absence of *AtNHX1* transcript in WT plants implying stress inducible expression of *AtNHX1*, which was consistent with salt tolerance in transgenic lines. However, significantly higher accumulation of *AtNHX1* transcripts in roots compared to leaves, implied enhanced activity of Na^+^/H^+^ antiporter in roots promoting active accumulation of Na^+^ in roots which in turn limited the transport of ions to shoots.

The transgenic lines grew well, flowered normally, and set pods and viable seeds even under 200 mM NaCl salt stress (Table S4). Although no significant difference was observed among different transgenic lines for plant height, branch number, pod number, seed number, seed weight per plant, and 10-seed-weight and length under 200 mM NaCl stress conditions, however, the WT plants showed significant reduction in these yield related traits under similar growth condition (Table S4). The WT plants displayed severe chlorosis, withered, and eventually died after 4 weeks of salt stress.

Plant yield-related parameters indicated that transgenics performed significantly better than WT and showed optimal vegetative growth and yield at high salt-stress (200 mM NaCl). The growth and yield response indicated that transgenics made better use of salt stress alleviation mechanism than WT possibly due to stress inducible expression of *AtNHX1*.

## Conclusion

In conclusion, transgenic mungbean that co-expressed *AtNHX1* and *bar* demonstrated enhanced tolerance to salinity, osmotic and oxidative stress under high salt stress due to inducible expression of *AtNHX1*, and herbicide tolerance. The overexpression of *AtNHX1* improved plant growth and yield through enhanced sequestration of ions into the vacuoles of transgenic roots and reduced transport of toxic Na^+^ to shoots thereby alleviating the toxic effect of Na^+^. Enhanced activity of vacuolar antiporter in transgenic plants was accompanied with restored water uptake through accumulation of compatible osmolytes, and membrane protection and unhampered photosynthesis through reduced generation and efficient scavenging of ROS by ascorbate and antioxidative enzymes. However, field trials are needed for further evaluation of salt stress and herbicide tolerance of these transgenic mungbean plants. The simultaneous increase of tolerance to salinity and herbicide in mungbean laid a strong foundation for stacking multiple traits of interest in this recalcitrant crop.

## Author contributions

LS conceived and designed the experiments. SK produced the transgenics and performed molecular analysis. SK performed physiological and biochemical experiments of transgenic plants. AK and RS assisted in molecular analysis. SK and LS critically analyzed the data and LS contributed to the writing of the manuscript.

### Conflict of interest statement

The authors declare that the research was conducted in the absence of any commercial or financial relationships that could be construed as a potential conflict of interest.
