# Supplementary material for: Co-expression of Arabidopsis NHX1 and bar Improves the Tolerance to Salinity, Oxidative Stress, and Herbicide in Transgenic Mungbean
Source: Front Plant Sci. 2017 Nov 2;8:1896. doi: 10.3389/fpls.2017.01896 (PMC5673651; doi:10.3389/fpls.2017.01896)
Supplement: Supplementary file 1 [file Table1.docx]

**Supplementary Table 1** Sequences of primers used for the study

| **Name and purpose** | **Primer name** | **Sequence(5’→3’)** |
| --- | --- | --- |
| *bar* for transgenic plants screeing | *bar*Fw | ACA GCG ACC ACG CTC TTGAA |
|  | *bar* Rv | TGC ACC ATC GTC AAC CAC TA |
| *AtNHX1* full CDS for transgenic plants screening | *AtNHX1* Fw | TGGAGACAATTTGATGACTC |
|  | *AtNHX1* Rv | TCAAGCCTTACTAAGATCAGG |
| Mungbean housekeeping for internal control | *Vu-tubulin*Fw | ACTGCATCTGCTATGTTCAG |
|  | *Vu-tubulin* Fw | GAATATCACACACACTCGAC |
| *AtNHX1* Expression analysis | *AtNHX1* partial Fw | AGCTACCTATTACCGCACCAGAACG |
|  | *AtNHX1* partial Rv | GCGAAACCCTATAAGAACCCTAATTCC |
