# Supplementary material for: Co-expression of Arabidopsis NHX1 and bar Improves the Tolerance to Salinity, Oxidative Stress, and Herbicide in Transgenic Mungbean
Source: Front Plant Sci. 2017 Nov 2;8:1896. doi: 10.3389/fpls.2017.01896 (PMC5673651; doi:10.3389/fpls.2017.01896)
Supplement: Supplementary file 2 [file Table2.docx]

**Supplementary Table 2** PCR conditions used for analysis

| **Name of primer** | **Primary denaturation** | **Secondary denaturation** | **Primer annealing** | **Initial extension** | **Final extension** | **No. of cycle repeat** |
| --- | --- | --- | --- | --- | --- | --- |
| *bar* | 95°C/ 4 min. | 95°C/ 1 min. | 58°C/ 30sec. | 72°C/ 1 min. | 72°C/ 5 min. | 35 |
| *AtNHX1* full CDS | 95°C/ 4 min. | 95°C/ 1 min. | 53°C/ 1 min.. | 72°C/ 1 min. | 72°C/ 5 min. | 35 |
| *AtNHX1* partial CDS | 95°C/ 4 min. | 95°C/ 1 min. | 58°C/ 45sec. | 72°C/ 1 min. | 72°C/ 10 min. | 35 |
| *Vr-tubulin* | 95°C/ 4 min. | 95°C/ 30 sec. | 54°C/ 30sec. | 72°C/ 30sec. | 72°C/ 5 min. | 35 |
|  |  |  |  |  |  |  |
