# Supplementary material for: Co-expression of Arabidopsis NHX1 and bar Improves the Tolerance to Salinity, Oxidative Stress, and Herbicide in Transgenic Mungbean
Source: Front Plant Sci. 2017 Nov 2;8:1896. doi: 10.3389/fpls.2017.01896 (PMC5673651; doi:10.3389/fpls.2017.01896)
Supplement: Supplementary file 3 [file Table3.docx]

**Supplementary Table 3:** Summary of *Agrobacterium tumefaciens –* mediated transformation and generation of *AtNHX1-bar* transgenic mungbean plants.

| **Exp. no.** | **No. of explants inoculated in Agrobacterium suspension** | **Shoot recovery on selection (%)** | **Number of plants rooted** | **No. of plants positive for *Atnhx1* and *bar* genes by PCR** | **Transformation efficiency^a^ (%)** |
| --- | --- | --- | --- | --- | --- |
| 1 | 85 | 48.23 | 5 | 2 | 2.35 |
| 2 | 90 | 48.18 | 8 | 3 | 3.34 |
| 3 | 110 | 55.11 | 6 | 4 | 3.63 |
| 4 | 120 | 46.34 | 5 | 3 | 2.5 |
| 5 | 105 | 47.97 | 4 | 2 | 1.9 |
| 6 | 98 | 35.71 | 5 | 2 | 2.04 |
| **Total /average** | **608^b^** | **48.36^c^** | **33** | **16^b^** | **2.62^c^** |

^a^ No of T_0_ plants PCR positive for *bar* and *Atnhx1*devided by the total no of explants co-cultivated

^b^ Total,

^c^Average
