# Supplementary material for: Co-expression of Arabidopsis NHX1 and bar Improves the Tolerance to Salinity, Oxidative Stress, and Herbicide in Transgenic Mungbean
Source: Front Plant Sci. 2017 Nov 2;8:1896. doi: 10.3389/fpls.2017.01896 (PMC5673651; doi:10.3389/fpls.2017.01896)
Supplement: Supplementary file 4 [file Table4.docx]

**Supplementary Table 4:** **Agronomic characteristics of WT and *AtNHX1-bar* transgenic mungbean lines**

| **Parameter** | **WT** | | **T_2_ *AtNHX1-bar* transgenic mungbean lines** | | | | |
| --- | --- | --- | --- | --- | --- | --- | --- |
|  |  |  | **CT_2_.9** | **AT_2_.1** | **ET_2_.4** | **DT_2_.2** | **CT_2_.3** |
|  | **200 mM NaCl stress** | **0 mM**  **stress** | **0 mM**  **NaCl stress** | **200 mM**  **NaCl stress** | | | |
| **Plant height (cm)** | 41 ± 1.15 | 55.3 ± 1.66 | 56.3 ± 1.20 | 46 ± 1.15 | 49.3 ± 2.72 | 54.3 ± 3.17 | 50 ± 1.52 |
| **Branch number** | 8.3 ± 0.88 | 10.6 ± 0.88 | 12.6 ± 0.33 | 09 ± 1.15 | 12 ± 1.52 | 13 ± 1.15 | 12.3 ± 0.88 |
| **Pod number/Plant** | 03 ± 0.57 | 09 ± 0.57 | 11.6 ± 0.88 | 5.6 ± 0.88 | 6.3 ± 0.88 | 07 ± 0.57 | 07.6 ± 0.88 |
| **Seed number/Plant** | 11.6 ± 2.02 | 47 ± 3.21 | 56.3 ± 5.7 | 36.3 ± 7.21 | 44 ± 4.16 | 46.3 ± 3.28 | 57 ± 9.29 |
| **Seed weight (mg)/Plant** | 49.3 ± 5.78 | 1644.7 ± 124.42 | 1937.7 ± 49.4 | 1171.7 ± 189.4 | 1277.3 ± 149.1 | 1438.3 ± 62.07 | 1745 ± 244.7 |
| **10 Seed weight (mg)** | 45 ± 3.78 | 314 ± 5.19 | 330 ± 6.80 | 341.6 ± 6.93 | 328.6 ± 8.68 | 324 ± 7.76 | 352.6 ± 6.06 |
| **10 Seed length (cm)** | 3.3 ± 0.11 | 4.6 ± 0.88 | 4.5 ± 0.08 | 4.6 ± 0.11 | 4.8 ± 0.05 | 4.4 ± 0.17 | 4.9 ± 0.14 |
