# Supplementary material for: Co-expression of Arabidopsis NHX1 and bar Improves the Tolerance to Salinity, Oxidative Stress, and Herbicide in Transgenic Mungbean
Source: Front Plant Sci. 2017 Nov 2;8:1896. doi: 10.3389/fpls.2017.01896 (PMC5673651; doi:10.3389/fpls.2017.01896)

Supplementary Figure:

Figure 1 Generation of transgenic mungbean plants. (A) Untransformed cotyledonary node explants on PPT selection medium (bar 1 mm); **(B)** Multiple shoot bud initiating from co-cultivated cotyledonary node explants on PPT selection medium (bar 1 mm); **(C)** Shoot elongation on PPT selection medium (bar 5 mm); **(D)** Rooting (bar 2 cm); **(E)** Hardening of plant in pot (bar 10 cm); **(F)** Flowering and pod formation in plants (bar 10 cm); and **(G)** T_2 hardened plants grown in pot under containment facility.

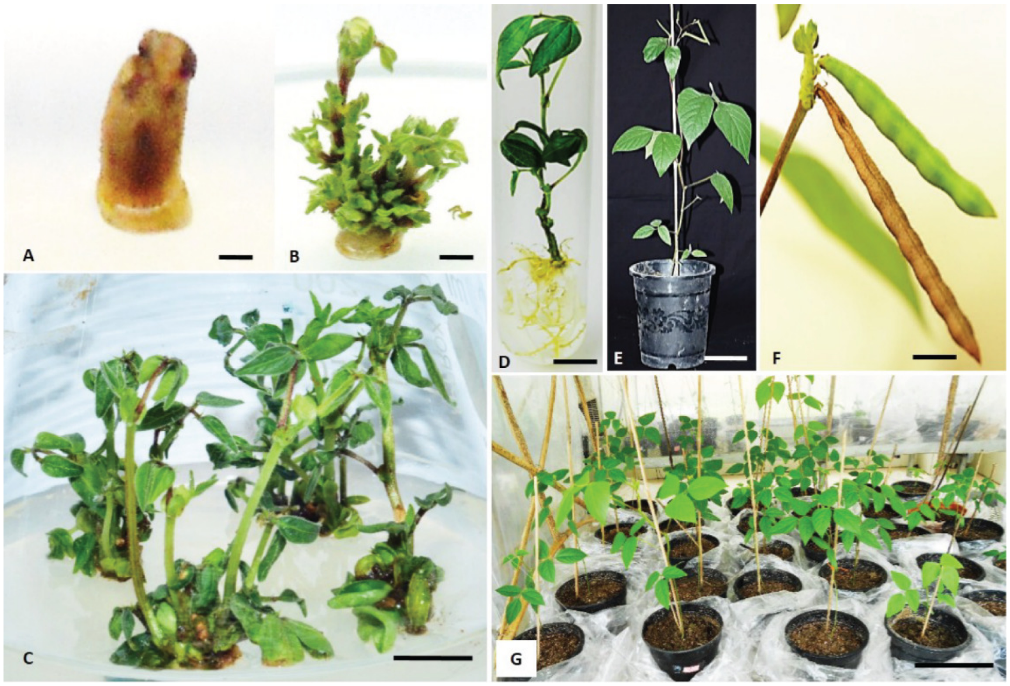

Supplement: Supplementary file 5 [file Presentation1.pdf]
